# Quality-by-Design Approach for the Development of Nano-Sized Tea Tree Oil Formulation-Impregnated Biocompatible Gel with Antimicrobial Properties

**DOI:** 10.3390/pharmaceutics12111091

**Published:** 2020-11-13

**Authors:** Thabata Muta, Ankit Parikh, Krishna Kathawala, Hanif Haidari, Yunmei Song, Jackson Thomas, Sanjay Garg

**Affiliations:** 1 Pharmaceutical Innovation and Development Group (PIDG), UniSA Clinical & Health Science, University of South Australia, City West Campus, North Terrace, Adelaide, SA 5000, Australia; Thabata.Muta@unisa.edu.au (T.M.); Ankit.Parikh@unisa.edu.au (A.P.); krishna.kathawala@unisa.edu.au (K.K.); hanif.haidari@mymail.unisa.edu.au (H.H.); May.Song@unisa.edu.au (Y.S.); 2 Faculty of Health, University of Canberra, Canberra, ACT 2617, Australia; Jackson.Thomas@canberra.edu.au

**Keywords:** tea tree oil, lipid-based nanotechnology, topical drug delivery, quality by design, mixture experimental design, controlled release, accelerated stability assessment, anti-microbial activity, *Staphylococcus epidermidis*, *Pseudomonas aeruginosa*

## Abstract

Despite the promising properties of tea tree oil (TTO) as potential therapeutics for several superficial skin conditions, certain limitations such as physical instability and skin irritation have restricted its widespread use. This study focuses on developing a rationally designed lipid-based nanoformulation (TTO-LNF) in accordance with the US Food and Drug Administration standard using a well-recognized quality-by-design (QbD) approach. Using a mixture experimental design, TTO-LNF has been optimized with 5% TTO, 10% surfactant, 5% co-surfactant, and 80% water, which showed a 14.4 ± 4.4 nm droplet size and 0.03 ± 0.01 polydispersity index (PDI). To ease the topical administration, the TTO-LNF gel formulation was further developed using xanthan gum to achieve the desired viscosity and form a gel. The in vitro antibacterial tests of TTO-LNF showed promising inhibitory effects toward both Gram-negative and Gram-positive bacteria. In fact, a complete growth inhibition of *S. epidermidis* was observed when exposed to TTO-LNF and TTO-LNF gel for 24 h, showing better activity than antibiotic kanamycin (25 µg/mL). Additionally, the in vitro release study showed a sustained release profile with a 50% release in 24 h, which could be beneficial to reduce the toxicity and thereby improve the therapeutic efficacy for long-acting applications. Furthermore, the formulations were remarkably stable at 40 °C/75% Relative humidity (RH) for at least 4 weeks. Therefore, this study presents a promising strategy to develop a biocompatible and stable formulation that can be used for the topical treatment of skin infections.

## 1. Introduction

Tea tree oil (TTO) is a volatile essential oil derived from the leaves of *Melaleuca alternifolia*, which is a native plant from Australia [1]. It is used as a treatment for a variety of conditions including acne, arthritis, burns, vaginal thrush, tinea, and dandruff due to its beneficial therapeutic properties such as antimicrobial, antiseptic, anti-inflammatory, and analgesic [2]. In particular, the antibacterial properties of TTO have recently gained strong research interest for the topical treatment of wound infection [3]. Studies have shown the promising efficacy of TTO products to decolonize bacteria against a range of superficial infections [4]. The exact mechanism of its antibacterial potential is not fully elucidated, but recent in vitro studies have shown broad-spectrum antibacterial activity against both Gram-negative and Gram-positive bacteria [5]. The disruption of the vital function of bacteria could happen as hydrocarbons can easily penetrate into the biological membrane, which may cause lysis, the loss of membrane integrity, leakage of ions, and an inhibition of respiration [6,7].

The chemical composition of TTO is well recognized and includes terpene hydrocarbons, monoterpenes, sesquiterpenes, and their associated alcohols. The volatile oil feature of TTO is due to the terpenes, and 90% of TTO evaporates fast from the epidermis, interfering with its absorption [8]. Terpinen-4-ol is the primary active component with concentration ranging between 30 and 48% [2]. Terpinen-4-ol is known to have antibacterial properties, but its activity depends on its ability to retain its stability and release at a sufficient rate. This suggests the importance of an optimized formulation that can retain the beneficial properties of TTO. Other components such as α-terpinene, γ-terpinene, and terpinolene can oxidize in the presence of high temperatures, atmospheric oxygen, light, and humidity. Their degradation causes skin irritation, allergic reactions, odor, and color changes [2,8,9]. The stability of TTO formulation remains a major research challenge that requires attention.

Lipid-based nanoformulations (LNF), specifically microemulsion, is composed of oil, surfactant with or without a co-surfactant, and water in droplet sizes that range between 10 and 100 nm [10]. They have the potential to reduce adverse effects (toxicity and irritation) by providing protection against volatilization and improving physical stability. They can also increase the therapeutic efficacy by increasing the solubilization of TTO, controlling the release and its distribution at the action site [10,11]. Additionally, the nano-sized droplet can also improve absorption by the skin [12].

TTO incorporated in nanocapsules has been reported in the literature [13,14,15]. However, the major drawbacks of the reported formulations are the presence of harsh solvents and chemicals that could potentially cause adverse effects, which is consistent with the USP <467> requirements [14,16]. Furthermore, the existing research uses the concentration of excipients beyond the limit specified by regulatory agencies such as US Food and Drug Administration for topical use [13,14,15]. Thus, the commercial translation of other reported topical formulations loaded with TTO is not possible [14,15,17,18,19]. To embrace the beneficial properties of TTO, it is imperative to rationally design a biocompatible formulation that can retain the inherent properties of TTO and simultaneously improve its biological activity.

As TTO is very sensitive to degradation in the presence of high temperatures, atmospheric oxygen, light, and humidity, the microemulsion-based strategy has been used in the present manuscript over nanoemulsion, because of its spontaneous formation ability without using any external energy and complicated manufacturing conditions. The nanoemulsion-based strategy requires specific manufacturing conditions, such as high pressure and extreme low and high temperatures [13,20,21,22]. Thus, the concentrations of each component including surfactant and co-surfactant in the development of LNF were considered critically. As such, the concentrations were determined in accordance with the amounts approved by the regulatory agencies, for example, the US Food and Drug Administration. Furthermore, formulation droplet size > 200 nm and polydispersity index (PDI) > 0.2 have shown low kinetic stability and were visually cloudy [11,14,23,24]; thus, based on the literature, parameters such as <200 nm droplet size and <0.2 PDI were considered as criteria for selecting surfactant and co-surfactant in the development of LNF [25].

To achieve the desirable characteristics of LNF, we used a quality-by-design (QbD) approach to understand how the process can affect the final product. QbD provides manufacturing controls that allow us to identify and optimize the process and formulation, according to the statistical experimental designs such as response surface [26]. This convenient process allows us to achieve high-quality specifications by increasing the process capability and manufacturing efficiencies (spontaneous emulsification with less surfactant and co-surfactant). In addition, QbD reduces the product variability [27,28]. The statistical experimental design, in particular, the Mixture Experimental Design, is an efficient method for batch selection and helpful for pharmaceutical formulation development and optimization. This is because it minimizes the number of experiments, correlates the independent variables (amount of water, surfactant, and co-surfactant) with the dependent variables (particle size and PDI), and expresses them in the mathematical model(s) to maintain the relation between the product variable with the required characteristics.

In this study, we conducted the preliminary screening of a potential surfactant and co-surfactant by evaluating their potential for forming nanoformulations with a droplet size < 200 nm and PDI < 0.2 [29]. Furthermore, we optimized the LNF with Design-Expert software (Design-Expert^®^ software version 11.1.0.1; Stat-Ease, Inc., Minneapolis, MN, USA). A Mixture Experimental Design was used to determine the effects of the three independent variables (surfactant, co-surfactant, and water) on the formulation parameters, including droplet size and PDI. TTO concentrations of 5–10% have been selected based on previous studies [30,31,32]. The amounts of surfactant and co-surfactant were considered within an approved limit by the regulatory agencies. Afterwards, we developed TTO-LNF gel, and extensive characterizations were carried out to ensure that it is an acceptable formulation with the appropriate viscosity and stability. We also performed an in vitro release study to monitor the release profile of the TTO from the formulation over time and predict its biological activity. The TTO formulation was also tested for antibacterial activity against both Gram-negative and Gram-positive bacteria.

## 2. Materials and Methods

### 2.1. Materials

TTO was purchased from Thursday Plantation, Qld, Australia. Tween-80 from Chem-supply (Gillman, Australia), Kolliphor™ RH40 from Sigma-Aldrich (St. Louis, MO, USA), Hydroxy Ethyl Cellulose (HEC) from Medicas (Plattsburgh, NY, USA), Xanthan Gum (Xantural 180^®^) from CP Kelco (Atlanta, GA, USA), and Carbopol^®^ 974P from Lubrizol (Cleveland, OH, USA) were obtained. High-performance liquid chromatography (HPLC) grade Methanol was obtained from EMD Millipore^®^ (Billerica, MA, USA) and the primary reference standard, terpinen-4-ol, was from Sigma Aldrich (Castle Hill, NSW, Australia). A Sartorius Ultra-Pure Water System was utilized in all studies (Goettingen, Lower Saxony, Germany). Ethanol was purchased from Thermo Fisher Scientific (Melbourne, Victoria, Australia). Transcutol P was given as a gratis sample from Carst & Walker Australia (Melbourne, Victoria, Australia).

### 2.2. Analytical Method for Characterization of TTO

The High-Performance Liquid Chromatography (HPLC) method was used with minor modification based on the literature [25,29,30]. As terpinen-4-ol is the major component of TTO, it has been used as the primary reference standard [2]. The analysis was conducted with an isocratic method using a PhenoSphere-NEXT™ 5µm C18 120 (250 × 4.6 mm) analytical column (Phenomenex, Torrance, CA, USA) at 40 °C, which is connected to an HPLC system (Shimadzu Corporation, Kyoto, Japan) consisting of a Diode Array Detector (SPD-M20A), an online degasser (DGU-20A3), a system controller (CBM-20A), an autosampler (SIL-20AHT), a pump (LC20AD), and an LC solution Chromopac data processor [33,34]. The mobile phase was a mixture of methanol and water (90:10, *v/v*) eluted at a flow rate of 1.0 mL/min. The sample injection volume was 20 µL, and the wavelength of 202 nm was selected for UV detection of terpinen-4-ol’s peak.

### 2.3. Preliminary Study for Selection of Critical Parameters and Limits for Mixture Experimental Design

This study was conducted with the selected therapeutic concentration of TTO (5%) based on the publication [30]. The preliminary screening of potential surfactant and co-surfactant were conducted in order to generate a nanoformulation with a droplet size < 200 nm and PDI < 0.2. Various surfactants, co-surfactants, and water with different ratios were considered (Table S1). The formulations containing Kolliphor™ RH40 and Tween-80 as surfactants and Transcutol P as a co-surfactant fulfilled all the required characteristics, such as droplet size (<200 nm) and PDI (<0.2). Thus, formulations were further optimized by Mixture Experimental Design to figure out the best concentrations and combination, based on the droplet size and PDI [35].

### 2.4. Preparation of LNF

**Figure 1:**
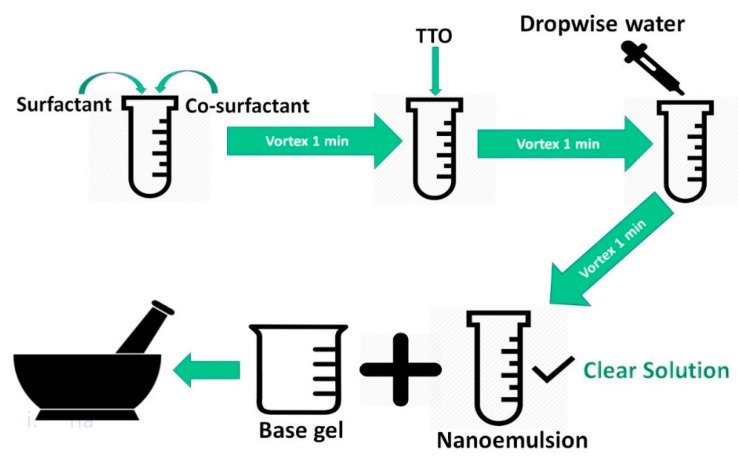
Lipid-based nanoformulations (LNF) development and incorporation into a base gel.

Transcutol P was mixed with Kolliphor™ RH40 and Tween-80 (50:50) using a VM1 vortex mixer (Ratek Instruments, Boronia, Victoria, Australia) for 1 min at 2700 RPM. Then, TTO (5% *w/w*, 50 mg/gm) was added and mixed, followed by the addition of water until a transparent emulsion was formed (Figure 1). The mixture was allowed to stabilize at room temperature (22 ± 0.5 °C) for approximately 45 min [29].

### 2.5. LNF Formulation Optimization Using Mixture Experimental Design

Based on the preliminary study, different proportions of surfactant (5–20% *w/w*), co-surfactant (5–50% *w/w*), and water (25–80% *w/w*) were investigated based on the effects on droplet size and PDI. The total sum of the percentage of these components and the amount of TTO (5%) was 100%. Moreover, the concentration of each component was chosen based on the safety profile requirement as per US Food and Drug Administration (Table 1) [36,37].

The Design-Expert software selected 14 randomized candidate points according to the parameters intended, including factorial points, centers of edges, axial checkpoints, overall center point, and constraints on plane centroids and each factor.

The model was chosen according to the variance analysis (ANOVA), standard deviation (SD), and the multiple determination coefficient (R^2^) value. Furthermore, to assure the effectiveness of each variable, ANOVA results should have a confidence level α ≤ 0.05. The predicted residual sum of squares should have a small value, a probability value (*p*-value) *p* ≥ 95%, and it is expected to have higher values of adjusted R^2^ and predicted R^2^. To assume effectiveness, adjusted R^2^, and predicted R^2^ should not have a gap of more than 0.2 [38].

### 2.6. Characterization of LNF: Droplet Size and PDI Measurement

#### 2.6.1. Zetasizer

The testing samples were diluted with water at 1:10 proportion before testing. The samples were sonicated (Bransonic; Danbury, CT, USA) for 10 s to prevent bubbles from interfering in the observation of droplet size and PDI [29]. The droplet size and PDI of TTO-LNF formulations were determined by a Dynamic Light Scattering instrument (Zeta sizer Nano, ZS90; Malvern Instruments Ltd., Malvern, UK) at 25 °C.

#### 2.6.2. Transmission Electron Microscopy (TEM)

The morphology of the TTO-LNF was investigated using a Tecnai G2 Spirit TEM (FEI Company, Hillsboro, OR, USA) operating at 100 KV. Samples were spotted on previously cleaned formvar/carbon 200 mesh grids using GATAN Solarus 950 Advanced Plasma Cleaner (Gatan Inc., Pleasanton, CA, USA) and left to adhere for 2 min. A piece of filter paper was utilized to remove the excess of liquid. Then, the grids were quickly washed with two drops of water and stained with 2% uranyl acetate (2 min) and allowed to dry for 5 min. The grid was loaded onto a specimen holder for analysis using the FEI Xplore3D software TEM (FEI Company, Hillsboro, OR, USA).

### 2.7. Development of LNF Gel

Step 1: The blank gel base was prepared by dissolving the selected polymers in water using a magnetic stirrer at 2700 RPM for 24 h.

Step 2: Transcutol P was mixed with Kolliphor™ RH40 and Tween-80 (50:50) using a VM1 vortex mixer (Ratek Instruments, Boronia, Victoria, Australia) for 1 min at 2700 RPM. Then, TTO (5% *w/w*, 50 mg/gm) was added and mixed with the above mixture.

Step 3: LNF gel was obtained by mixing vehicle mixture obtained from step 2 with blank gel from step 1 in the volume ratio of 1 to 4, under gentle mixing using a mortar and pestle (Figure 1).

The tested polymers were xanthan gum 1.25% (*w/w*), hydroxyethyl cellulose (HEC) varied into 1.0% to 3.0% (*w/w*) and Carpobol^®^ 974P varied into 0.6–1.0% (*w/w*). The optimized formulation has been developed using xanthan gum 1.25% (*w/w*). The consistency of LNF gel has been matched with the desired viscosity of a well-accepted marketed topical preparation “Dove silky nourishment body cream (Unilever, Wirral, UK)”. The droplet size and PDI of LNF gel have been measured using the protocol mentioned in Section 2.6.1.

### 2.8. Characterization of LNF Gel

#### 2.8.1. Viscosity Measurements

The viscosity of each formulation was measured in triplicate by Rheosys Merlin VR (Scientex Pty Ltd., Melbourne, Victoria, Australia) with a 15 mm parallel plate, a 0.5 mm gap between the equipment and the plate, and a temperature-controlled parallel plate set at 25 °C with 15 s of thermal equilibrium. The pre-shear speed was constant at 10 (1/s) for 20 s. The delay time of 60 s and integration time of 5 s was followed by the speed oscillation in 10 steps starting from 1 (1/s) to 100 (1/s).

#### 2.8.2. In Vitro Release Study

Franz diffusion cells (specially designed, 20 mL of receptor volume, and 1.77 cm^2^ of diffusion area) were submerged in a water bath (Ratek WB14, Boronia, Victoria, Australia) set at 32 °C. The acceptor compartment was filled with release media and an Isopore membrane with 0.22 µm pore diameter (Millipore, Bedford, MA, USA) positioned between the donor and acceptor compartments [39]. The release media (ethanol/water, 50/50, *v/v*;) was mixed with a magnetic stirring bar, TTO-LNF gel (2 g) or TTO-LNF (2 g) was added on the membrane respectively, and the compartments were sealed with Parafilm (Bemis^®^, Neenah, WI, USA). The cells and the dissolution media were kept in the water-jacket at 32 °C. Then, 0.8 mL of sample was collected from the acceptor compartment and replaced immediately with the same volume of the fresh dissolution media at the pre-determined time points. The samples collected at 0.5, 1.0, 1.5, 2.0, 4.0, 6.0, 12.0, and 24.0 h were analyzed with the developed PDA-HPLC method without dilution [15,23,40].

#### 2.8.3. Stability Study

TTO-LNF gel in a glass vial container was stored at 4 ± 1 °C and 40 ± 1 °C for 4 weeks, respectively. The accelerated condition has been selected to predict a longer-term stability. All stability studies were carried out with 3 batches and observed in terms of physical appearance, homogeneity, globule size, and PDI. Finally, the samples were collected and analyzed for terpinen-4-ol concentration using the developed PDA-HPLC method.

### 2.9. In Vitro Antibacterial Evaluation of LNF Formulations

#### 2.9.1. Bacterial Strains and Culture

Representative strains of Gram-negative and Gram-positive bacteria were used. Bacterial strains of *Staphylococcus epidermidis* ATCC 35984 and *Pseudomonas aeruginosa* PAO1 were sourced from −80 °C and were selectively grown on Tryptic Soy Agar (TSA) plates (Thermo Fisher, Waltham, MA USA). Overnight bacterial cultures were prepared by isolating a single colony and suspending it in 10 mL sterile Tryptic Soy Broth (TSB) medium (Thermo Fisher, Waltham, MA USA). The bacterial culture was incubated overnight (16–18 h) at 37 °C in a shaking incubator (Adelab Scientific, Adelaide, SA, Australia). The next day, the optical density of bacterial culture was measured at 660 nm (OD660) and standardized to 0.25, which equates to approximately 2.5 × 10^8^ CFU/mL.

#### 2.9.2. Well Diffusion Assay

The good diffusion assay was employed to test the susceptibility of TTO-LNF formulations against different bacterial strains. The method was employed based on our recent study and in accordance with the Clinical and Laboratory Standards Institute (CLSI) [41]. Firstly, bacterial strains of (*S. epidermidis* and *P. aeruginosa*) were grown overnight at 37 °C in TSB, and then its concentration was adjusted to OD 660 nm 0.25. Thereafter, it was further diluted to obtain 1 × 10^6^ CFU mL in TSB. Then, the bacterial lawn was prepared on the surface of the agar plate by adding 100 μL of diluted inoculum and spread uniformly using a sterile cotton swab. Then, using a sterile cork borer, a well with a diameter of 6 mm was punched aseptically (6 wells/plate). The test formulations and positive control (Kanamycin 25 µg/mL) were introduced into the well (100 µL). The agar plates were transferred to the 37 °C incubator overnight (16–18 h) and the zone of inhibition (mm) was measured the following day. Each test was carried out at least in triplicates.

#### 2.9.3. Time Killing Curve

The time kill curve was performed according to our previously published method with a slight modification [42]. This assay was employed to determine the pattern of bacterial growth or killing kinetics of the TTO formulation against representative Gram-positive bacteria (*S. epidermidis*). This strain was used for its known presence in common wound infection. The test organism was grown overnight, and its optical density was adjusted to OD 660 nm 0.25 and further diluted 1:100 in TSB. The effect of different TTO formulations on test organisms was studied using TTO formulations, the blank formulations alongside an appropriate positive control (Kanamycin) and negative control (TSB). Briefly, a bacterial suspension 100 µL (1 × 10^6^ CFU/mL) was added into all wells except for the sterility control blank. Then, 100 µL of the formulations at different concentrations were added to the wells, and the time zero absorbance was measured. Thereafter, the absorbance was measured every two hours using an automated multimode plate reader (PerkinElmer, Waltham, MA USA) at 37 °C for 24 h. The effect of TTO formulation was compared against the untreated control.

## 3. Results and Discussion

### 3.1. Analytical Method for Characterisation of TTO

The HPLC method was used to determine the peak area of each standard and to generate the calibration curve (Figure S1). The mean regression equation, for TTO for the concentration range of 25–150 µg/mL ran in triplicate, was y = 7705.6x + 56,070 (R^2^ = 0.9983, *n* = 6). In addition, the equation for the primary reference standard (terpinen-4-ol) was y = 21,036x + 170,236 (R^2^ = 0.9975, *n* = 5), where x represents the concentration and y represents the peak area of terpinene-4-ol. According to the mean regression equation, TTO from Thursday Plantation (Ballina, NSW, Australia) had 33.33% of terpinen-4-ol.

### 3.2. Preliminary Study for Selection of Ingredients, Critical Parameters, and Limits for Mixture Experimental Design

The selection of surfactants and co-surfactants was based on the literature for preliminary trials [30]. The results are shown in Table S1. The criteria such as droplet size < 200 nm and PDI < 0.2 were used as a screening tool for the selection of surfactants and co-surfactants (Table 1). The appropriate selection of surfactants was a crucial factor since it may cause skin irritation. The maximum limit of the concentration per unit for each ingredient as per the US Food and Drug Administration was considered as a critical factor in screening, such as propylene glycol (8% *w/w*), Tween-80 (15% *w/w*), Span-80 (7% *w/w*), Transcutol P (49.91% *w/w*), and Kolliphor™ RH40 (20% *w/w*) [43]. Non-ionic hydrophilic surfactants, such as Kolliphor™ RH40 and Tween-80, are considered less toxic than ionic surfactants [44]. Transcutol P was found to be nontoxic and biocompatible with the human skin [45]. They have been considered in this study, as they can also form more stable nanoemulsions, with less energy and surface activity required to form the emulsion [10]. Based on the preliminary screening, the mixture of Kolliphor™ RH40 and Tween-80 (50:50) as surfactants and Transcutol P as a co-surfactant have been selected.

### 3.3. Mixture Experimental Design for LNF Formulation Optimization

The composition of each batch and the response variables of TTO-LNF predicted by Mixture Experimental Design are presented in Table 2. The minimum values of droplet size and PDI are shown in the batch TTO003QbD1. Comparing the results of batch TTO003QbD1 with TTO003QbD5 and TTO003QbD6, it suggested that when the proportion of co-surfactant decreases while the amount of water increases, the globule size of TTO-LNF formulation tends to constrict. Furthermore, the same response was observed when the amount of surfactant increases and the amount of water decreases, as we found in batches TTO003QbD10, TTO003QbD12, TTO003QbD7, and TTO003QbD8. The same results were observed in the response surface analysis. For the interpretation, R^2^ and ANOVA were analyzed to validate the suitability of the regression model in the relation to a lack-of-fit test.

#### 3.3.1. Analysis of Variance 

**Figure 2:**
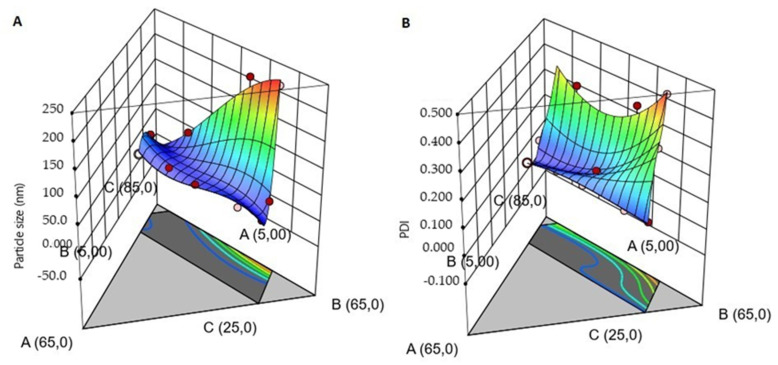
Three-dimensional surface plots interaction effect between the variables (Kolliphor™ RH40, Tween 80, Transcutol P and Water) on the response droplet size (**A**) and polydispersity index (PDI) (**B**).

The classical experimental designs require many factors and a fraction of the runs to produce a response. However, in this study, the response depends on the proportions, not the number of ingredients; therefore, the factorial designs were not considered, as it may not give a better prediction. Based on Table 3, the cubic model was selected for droplet size and PDI results according to the minimum SD value, as well as the higher values of adjusted R^2^ and predicted R^2^. The differences of the adjusted R^2^ and the predicted R^2^ values were 0.33 nm and 0.19 for the droplet size and PDI, respectively. Although the difference for droplet size was not in accordance to the desired range (0.2) and it may indicate a large block effect, the cubic model was still selected because it matches with the other criteria. According to Table 3, both responses had adjusted R^2^ > 0.9; thus, it suggested that the observed result was better than the predicted one. The “Adeq Precision” ratio is greater than 4, which is desirable and indicates an adequate signal for the responses. In conclusion, the cubic model was suitable to compare the interaction between the parameters, so the models can be interpreted by the graphical presentation (Figure 2).

#### 3.3.2. Response Surface Analysis

The droplet size decreases when the formulation has high surfactant concentration and low co-surfactant concentration (blue color in Figure 2). The same response has been observed for PDI; when the surfactant concentration is constant, the concentration of water increases and the co-surfactant decreases. Thus, it is possible to see a wide difference in the PDI values (<0.1), but it does not occur for the droplet size (e.g., batches TTO003QbD10 and TTO003QbD14). This is possibly due to the presence of a sufficient amount of surfactant to emulsify the TTO and the aqueous phase to create nano-sized droplets. These responses are because of the optimum quantity of surfactant and co-surfactant. Moreover, mixing with a vortex mixer could facilitate a faster adsorption of emulsifier to the oil droplet surface, which further lowers the interfacial tensions to generate nano-sized droplets. The interfacial tension can be decreased when the number of emulsifiers increases, generating a reduction in Laplace pressure and required stress for droplet deformation. Furthermore, the biggest droplet can be produced when there is more emulsifier than necessary to cover the droplet surfaces [38].

For the better clarification of each factor interfering with the response, we can analyze the *p*-value and the equation to predict and identify the relative impact of the factor by comparing its coefficient. In the equations, a positive sign for each factor shows a high effect on the mixture, and a negative sign shows the low impact of the component in the formulation [19].

#### 3.3.3. ANOVA for Cubic Model

A, B, and C, reported at Tables S2 and S3, represent the fractions of surfactant (Tween 80 and Kolliphor™ RH40, *w/w*, 50/50), co-surfactant, and water in %*w/w*, respectively. BC had the highest F-value and lowest *p*-value in both tables, which means that the co-surfactant with water had the greatest influence on the formulation.

#### 3.3.4. Final Equation in Terms of Real Components Verification of Models

Droplet size = −308.63794A + 2.09293B − 0.858838C + 6.41918A × B + 5.37673A × C + 0.200024B × C − 0.074004A × B × C + 0.046675A × B × (A − B) + 0.021627A × C × (A − C) + 0.001560B × C × (B − C)(1)

PDI = −1.40054A + 0.027821B + 0.014700C + 0.023319A × B + 0.025780A × C − 0.000535B × C − 0.000225A × B × C + 0.000108A × B × (A − B) + 0.000142A × C × (A − C) − 6.70458E-07B × C × (B − C)(2)

Overall, according to the equations, it can be concluded that all the mixture components (A, B, and C) had an impact on the predicted responses. However, conforming to the final mathematical equations with the highest value for factor B in both cases, the highest F-value and lowest *p*-value (<0.05) had the greatest influence on the droplet size, and PDI was shown by the factor B (Transcutol *p*). In conclusion, the graphics and the equation results are in concordance.

#### 3.3.5. Verification of Models Optimization

The verification was done to validate the actual results with the predicted values. The formulation showed 14.4 nm as droplet size and 0.06 as PDI predicted values. The actual obtained values were 14.4 nm ± 4.4 nm for droplet size and 0.03 ± 0.01 for PDI.

#### 3.3.6. Formulation Optimization

Based on software, the formulation composed of ingredients such as 5% TTO, 9.88% of surfactant (Kolliphor™ RH40:Tween-80 = 50:50, *w/w*), 5.11% of co-surfactant (Transcutol P), and 80% of water should pass the all desirable criteria. Thus, TTO-LNF was prepared using 5% TTO, 10% of surfactant (Kolliphor™ RH40:Tween-80 = 50:50 *w/w*), 5% of co-surfactant (Transcutol P), and 80% of water and considered for further characterization.

### 3.4. Characterization of LNF

The droplet size and the PDI value of the optimized formulation of TTO-LNF were found to be 14.4 ± 4.4 nm and 0.03 ± 0.05, respectively (Figure S2). In addition, TEM analysis confirmed the results obtained from the zeta sizer. Moreover, The TEM image of TTO-LNF demonstrated the spherical shape.

### 3.5. Screening of Polymers for the Development of TTO-LNF Gel

Different polymers were used for incorporating the TTO-LNF as reported in Table 4, but only xanthan gum showed physical compatibility, retaining a nano-size of LNF and transparency. The concentration of xanthan gum was optimized to achieve a lotion consistency. Xanthan gum offers better spreadability for the topical treatment of body parts which are difficult to treat—for example, the scalp and hirsute and intertriginous areas [46,47]. We considered a shear rate (1/s) of 21.441 and a mean viscosity (Pa.s) of 5.1070 from a Dove silky nourishment body cream (Unilever, Wirral, UK) as standard. The TTO-LNF gel with xanthan gum (1.25%) successfully matched the desired viscosity. Thus, the optimised TTO-LNF gel-based topical formulation was selected for further characterization.

### 3.6. Characterization of TTO-LNF Gel

#### 3.6.1. In Vitro Release Study

**Figure 3:**
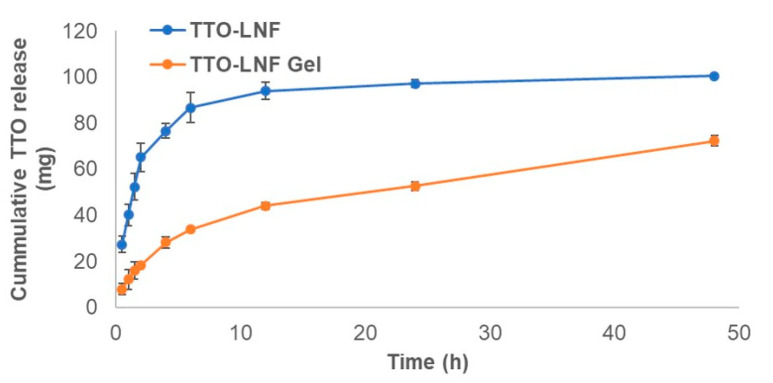
In vitro release of tea tree oil (TTO) from TTO-LNF gel and TTO-LNF.

The release study was performed to evaluate the release of TTO from TTO-LNF gel compared to the TTO-LNF. The release profiles of TTO-LNF gel and TTO-LNF are shown in Figure 3 as a function of time. The sustained release of TTO was observed with nearly 50% and 72% in 24 h and 48 h from TTO-LNF gel, respectively. About 100% TTO was released within 24 h from TTO-LNF. The controlled release for the topical formulation is more promising than the conventional options as it promotes better effectiveness and less toxicity [48]. Thus, we expect that the sustained release of TTO from TTO-LNF-based preparation would offer help in reducing the skin irritation as well as a long-term activity with improved permeation.

#### 3.6.2. Stability Study

The stability study was conducted on TTO-LNF gel under conditions at 4 °C and 40 °C/75% Relative humidity (RH) to investigate the effect of the temperature, as recommended by the International Conference on Harmonization (ICH) guideline. The difference in concentration of TTO was considered as a primary factor in stability assessment. Droplet size is a critical parameter; less than 200 nm is the expected value desired for high kinetic stability and high transmittance [12]. On the other hand, PDI values below 0.1 predict concordance, stability, and homogeneity [49]. The effect of temperature on LNF gel was found to be insignificant after 4 weeks of storage. All formulations were found to be stable (no phase separation); there was no change in odor, homogeneity, or syneresis (Table 5). The gelling matrix provides more protection and stability to TTO-LNF. Moreover, TTO-LNF showed >80% of TTO content at 40 °C storage for 4 weeks, which suggests higher protection for TTO in comparison to previous studies [50]. 

### 3.7. In Vitro Antibacterial Evaluation

**Figure 4:**
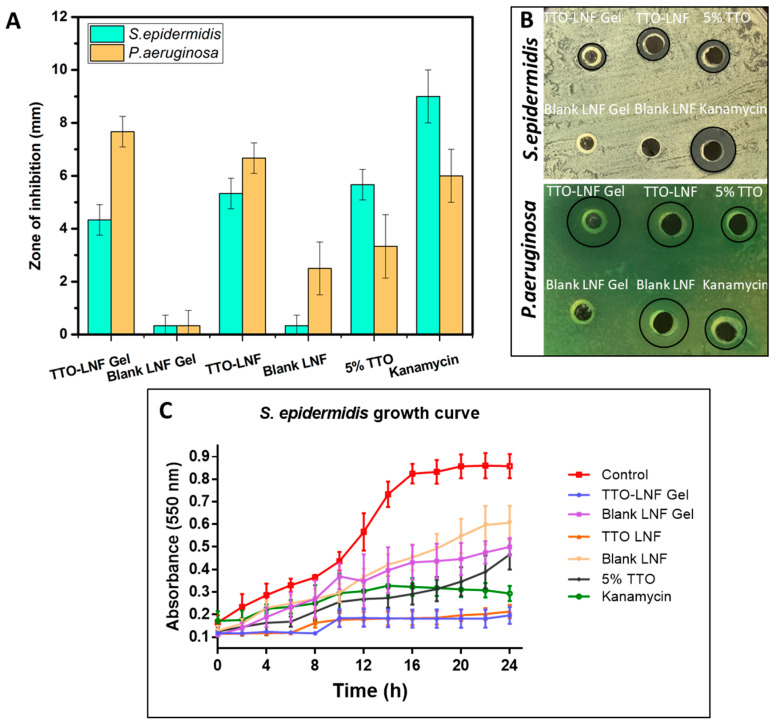
(**A**) *S. epidermidis* and *P. aeruginosa* zone of inhibition after treating with the different formulation and its corresponding representative digital photographs (**B**). (**C**) Time-dependent growth curve of *S. epidermidis* when treated with formulations in comparison to the untreated control.

The effect of TTO formulation was studied against representative both Gram-negative and Gram-positive bacteria, namely, *S. epidermidis* and *P. aeruginosa*. These bacteria are well known for their association in common wound infection [51]. The results of the zone of inhibition (ZOI) for both bacteria are shown in Figure 4A,B. The result showed a clear zone of inhibition for TTO-LNF gel, where *P. aeruginosa* (7.8 mm) is more susceptible than *S. epidermidis* (4.2 mm). Similarly, the TTO-LNF showed 6.4 mm for *P. aeruginosa* and 5 mm for *S. epidermidis*. Meanwhile, the blank for both formulations showed no inhibitory effect toward both bacteria (<1 mm). Interestingly, the TTO-LNF gel is shown to be more effective in killing bacteria than TTO nano, particularly with *P. aeruginosa.* This could be explained based on the difference in viscosity and release profile. The formulation was also shown to be more effective than the 5% TTO solution, suggesting that our formulation is not preventing the release of active compounds from the TTO and possibly maintaining its inherent biological properties. Based on the release data, nearly 50% (2.5 mg) and 100% (5 mg) of TTO should be available at 24 h for antibacterial activity from TTO-LNF gel and TTO-LNF formulations. However, it does not represent the actual situation, as there is no sink condition maintained in antibacterial experiments, and there is a significant difference between the composition and the amount of release media and bacterial culture media for both experiments. Therefore, it is very difficult to correlate the in vitro release with the antibacterial study.

Furthermore, the bacterial growth curve was conducted over time to monitor the growth kinetics of bacteria in the presence or absence of TTO formulations. As presented in Figure 4B, the untreated control shows a typical growth curve of *S. epidermidis* bacteria with increased absorbance (nm) after 12 h. In contrast, the treatment of *S. epidermis* with TTO-LNF gel and TTO-LNF results in a remarkable suppression of bacteria growth for 24 h. In fact, the TTO formulation was more effective to inhibit growth than an antibiotic, which is consistent with the ZOI assay. The blank gel had minimal impact on the growth of bacteria over time. There is antibacterial activity of Tween 80 against *Pseudomonas aeruginosa* [52] and *Staphylococcus epidermidis* reported [53]. Tween 80 (5%) has been used in our blank formulations, which could be the reason for antibacterial effects of blank LNF and blank LNF gel.

The result observed here shows the promising antibacterial potential of TTO formulation against Gram-negative and Gram-positive bacteria. Additionally, according to this assay, our formulations showed better antibacterial activity than kanamycin 25 µg/mL. However, further studies may be required to understand the antibacterial mechanism of TTO. Nevertheless, we have clearly shown the ability to incorporate TTO at the appropriate dose and maintain its stability and biological activity, as demonstrated here.

## 4. Conclusions

TTO-LNF was successfully developed using Mixture Experimental Design using a minimum required concentration of surfactant and co-surfactant. The selected TTO-NLF formulation was incorporated with 5% of TTO, 10% of surfactant (Kolliphor™ RH40:Tween-80 = 50:50, *w/w*), 5% of co-surfactant (Transcutol P), and 80% of water. The developed TTO-LNF demonstrated a droplet size of 14.4 ± 4.4 nm and PDI of 0.03 ± 0.05, which perfectly matched the predicted response value. TTO-LNF gel was developed using Xanthan gum in the present study with the characteristics consistent with the well-accepted market preparation. The sustained release profile of TTO from TTO-LNF based preparation will offer help in reducing the skin irritation as well as a long-term activity with the improved permeation. Additionally, the TTO-LNF showed the significantly improved physical stability of TTO. The antibacterial results showed that the testing formulations were effective toward bacteria by inhibiting their growth, and they were better than approved antibiotic (Kanamycin) and 5% TTO solution. The remarkable activity against bacteria is possibly attributed to its well-defined distribution in the formulation and sufficient release to kill bacteria. The present study demonstrates a significant advancement compared to previous TTO formulations. Therefore, this study presents a well-defined strategy to develop TTO-LNF formulations with acceptable pharmaceutical properties, and it thereby offers a promising candidate for a variety of skin infections and in the management of inflammatory/immune disorders affecting the skin.

## Figures and Tables

**Table 1:** Restrictions of variables and response on quality-by-design (QbD).

**Symbol**	**Variables**	**Units**	**Range (%)**	
			**Lower**	**Higher**
A	Surfactant (Kolliphor™ RH40: Tween 80, 50:50)	%, *w/w*	5	20
B	Co-surfactant (Transcutol P)	%, *w/w*	5	50
C	Water	%, *w/w*	25	80
**Symbol**	**Response**	**Units**	**Range (%)**	
			**Lower**	**Higher**
PDI	Polydispersity index	-	0.01	0.2
DS	Droplet size	nm	1	200

**Table 2:** Mixture Experimental Design batch and results.

Batch	Surfactant (%)	Co-Surfactant (%)	Water (%)	Droplet Size (nm)	PDI
TTO003QbD1	20	5	70	14.42	0.03
TTO003QbD2	5	40.0	50	203.5	0.32
TTO003QbD3	5	17.8	72.2	39.08	0.30
TTO003QbD4	15.5	36.5	43	16.74	0.11
TTO003QbD5	20	26.6	48.4	15.25	0.05
TTO003QbD6	19	16.3	59.7	14.85	0.05
TTO003QbD7	20	50	25	16.10	0.05
TTO003QbD8	5	50	40	211.66	0.41
TTO003QbD9	10	5	80	18.51	0.05
TTO003QbD10	12.6	28.9	53.5	17.41	0.07
TTO003QbD11	20	41.8	33.2	16.05	0.04
TTO003QbD12	5	27.4	62.6	94.45	0.19
TTO003QbD13	13.10	28.8	53.1	17.26	0.08
TTO003QbD14	12.1	50	32.9	21.01	0.26

**Table 3:** Selection of the cubic model.

**ANOVA**					
**Droplet Size**					
**Fit Statistics**	**Linear**	**Quadratic**	**Special Cubic**	**Cubic**	**Special Quartic**
SD	48.6	28.7	30.7	14.3	33
R^2^	0.587	0.895	0.895	0.987	0.913
Adjusted R^2^	0.511	0.829	0.805	0.957	0.775
Predicted R^2^	0.270	0.470	0.142	0.627	−1.59
Adeq. Precision	7.87	11.7	10.2	17.75	7.73
F-value	7.81	13.63	9.95	33.5	6.59
*p*-value	0.007	0.000964	0.00391	0.00206	0.0264
**PDI**					
SD	0.0622	0.0494	0.0456	0.0319	0.0506
R^2^	0.802	0.909	0.932	0.981	0.940
Adjusted R^2^	0.766	0.852	0.874	0.939	0.845
Predicted R^2^	0.654	0.667	0.484	0.744	−0.558
Adeq. Precision	13.3	12	13.3	14.6	10.6
F-value	22.3	16.0	16.0	23.0	9.87
*p*-value	0.000135	0.000550	0.000904	0.00424	0.0110

**Table 4:** Screening and optimization of polymers for LNF gel.

Batch	Formulation	% Polymer	Physical Appearance	Mean Viscosity (Pa.s) at 21.441 Shear Rate (1/s)
1	Hydroxyethylcellulose + LNF	1–3	Lotion/clear/2 layers	NM ^1^
2	Carbopol 974P + LNF	0.6–1	Gel/cloudy	NM ^1^
3	Silky nourishment body cream (Unilever)	-	Cream	5.72
4	LNF gel 5% TTO	1.25	Lotion/clear	5.76

^1^ NM: Not measured.

**Table 5:** Stability study of optimized TTO-LNF gel (mean ± SD, *n* = 3).

Sample	Droplet Size (nm)		PDI		TTO Concentration (µg/mL)	
	4 °C	40 °C	4 °C	40 °C	4 °C	40 °C
LNF gel day 0	33.4 ± 4.4		0.47 ± 0.05		79.3 ± 8.2	
LNF gel day 28	31.2 ± 4.8	25.2 ± 5.2	0.44 ± 0.03	0.44 ± 0.05	85.2 ± 6.3	63.8 ± 4.9

## Supplementary Materials

The following are available online at https://www.mdpi.com/1999-4923/12/11/1091/s1, Figure S1. PDA-HPLC chromatograms of TTO and primary reference standard (Terpinen-4-ol). Chromatogram for TTO at concentrations 50 µg/mL (A) and 100 µg/mL (B). Primary reference standard (Terpinen-4-ol) at 50 µg/mL (C), Retention time at 4.2 min, Figure S2. Characterization of optimized TTO-LNF formulation droplet size using zeta sizer (A), transmission electron microscopy with 500 nm scale (B), and 100 nm scale (C), Table S1. Preliminary screening of components for development of TTO-LNF, Table S2. ANOVA for droplet size and Cubic Model.

## References

[1] Kawakami: M., Sachs R.M., Shibamoto T. (1990). Volatile Constituents of Essential Oils Obtained from Newly Developed Tea Tree (Melaleuca-Alternifolia) Clones. J. Agric. Food Chem..

[2] De Groot A.C., Schmidt E. (2016). Tea tree oil: Contact allergy and chemical composition. Contact Dermat..

[3] Cox S.D., Mann C.M., Markham J.L., Bell H.C., Gustafson J.E., Warmington J.R., Wyllie S.G. (2000). The mode of antimicrobial action of the essential oil of Melaleuca alternifolia (tea tree oil). J. Appl. Microbiol..

[4] Hammer K.A., Carson C.F., Riley T.V. (2012). Effects of melaleuca alternifolia (tea tree) essential oil and the major monoterpene component terpinen-4-ol on the development of single- and multistep antibiotic resistance and antimicrobial susceptibility. Antimicrob. Agents Chemother..

[5] Dryden M.S., Dailly S., Crouch M. (2004). A randomized, controlled trial of tea tree topical preparations versus a standard topical regimen for the clearance of mrsa colonization. J. Hosp. Infect..

[6] Carson C.F., Hammer K.A., Riley T.V. (2006). Melaleuca alternifolia (tea tree) oil: A review of antimicrobial and other medicinal properties. Clin. Microbiol. Rev..

[7] Cox S.D., Mann C.M., Markham J.L., Gustafson J.E., Warmington J.R., Wyllie S.G. (2001). Determining the antimicrobial actions of tea tree oil. Molecules.

[8] Thomas J., Davey R., Peterson G.M., Carson C., Walton S.F., Spelman T., Calma T., Dettwiller P., Tobin J., McMillan F. (2018). Treatment of scabies using a tea tree oil-based gel formulation in australian aboriginal children: Protocol for a randomised controlled trial. BMJ Open.

[9] Pazyar N., Yaghoobi R., Bagherani N., Kazerouni A. (2013). A review of applications of tea tree oil in dermatology. Int. J. Derm..

[10] Azeem A., Rizwan M., Ahmad F.J., Iqbal Z., Khar R.K., Aqil M., Talegaonkar S. (2009). Nanoemulsion components screening and selection: A technical note. AAPS PharmSciTech.

[11] Flores F.C., de Lima J.A., Ribeiro R.F., Alves S.H., Rolim C.M., Beck R.C., da Silva C.B. (2013). Antifungal activity of nanocapsule suspensions containing tea tree oil on the growth of trichophyton rubrum. Mycopathologia.

[12] Sonneville-Aubrun O., Simonnet J.T., L’Alloret F. (2004). Nanoemulsions: A new vehicle for skincare products. Adv. Colloid Interface Sci..

[13] Sinha P., Srivastava S., Mishra N., Singh D.K., Luqman S., Chanda D., Yadav N.P. (2016). Development, optimization, and characterization of a novel tea tree oil nanogel using response surface methodology. Drug Dev. Ind. Pharm..

[14] Najafi-Taher R., Ghaemi B., Amani A. (2018). Delivery of adapalene using a novel topical gel based on tea tree oil nano-emulsion: Permeation, antibacterial and safety assessments. Eur. J. Pharm. Sci..

[15] Wulansari A., Jufri M., Budianti A. (2017). Studies on the formulation, physical stability, and in vitro antibacterial activity of tea tree oil (melaleuca alternifolia) nanoemulsion gel. Int. J. Appl. Pharm..

[16] Chang R.K., Raw A., Lionberger R., Yu L. (2013). Generic development of topical dermatologic products: Formulation development, process development, and testing of topical dermatologic products. AAPS J..

[17] Biju S.S., Ahuja A., Khar R.K., Chaudhry R. (2005). Formulation and evaluation of an effective ph balanced topical antimicrobial product containing tea tree oil. Pharmazie.

[18] Effendy I., Maibach H.I. (1995). Surfactants and experimental irritant contact dermatitis. Contact Dermat..

[19] Barot B.S., Parejiya P.B., Patel H.K., Gohel M.C., Shelat P.K. (2012). Microemulsion-based gel of terbinafine for the treatment of onychomycosis: Optimization of formulation using d-optimal design. AAPS PharmSciTech.

[20] Mishra B., Patel B.B., Tiwari S. (2010). Colloidal nanocarriers: A review on formulation technology, types and applications toward targeted drug delivery. Nanomedicine.

[21] McClements D.J. (2012). Nanoemulsions versus microemulsions: Terminology, differences, and similarities. Soft Matter.

[22] Marzuki N.H.C., Wahab R.A., Hamid M.A. (2019). An overview of nanoemulsion: Concepts of development and cosmeceutical applications. Biotechnol. Biotechnol. Equip..

[23] Reichling J., Landvatter U., Wagner H., Kostka K.H., Schaefer U.F. (2006). In vitro studies on release and human skin permeation of australian tea tree oil (tto) from topical formulations. Eur. J. Pharm. Biopharm..

[24] Sonia K., Anupama D. (2011). Microemulsion based transdermal drug delivery of tea tree oil. Int. J. Drug Dev. Res..

[25] Clayton K.N., Salameh J.W., Wereley S.T., Kinzer-Ursem T.L. (2016). Physical characterization of nanoparticle size and surface modification using particle scattering diffusometry. Biomicrofluidics.

[26] Oh G.H., Park J.H., Shin H.W., Kim J.E., Park Y.J. (2018). Quality-by-design approach for the development of telmisartan potassium tablets. Drug Dev. Ind. Pharm..

[27] Yu L.X., Amidon G., Khan M.A., Hoag S.W., Polli J., Raju G.K., Woodcock J. (2014). Understanding pharmaceutical quality by design. AAPS J..

[28] Kumar M., Bishnoi R.S., Shukla A.K., Jain C.P. (2019). Techniques for formulation of nanoemulsion drug delivery system: A review. Prev. Nutr. Food Sci..

[29] Parikh A., Kathawala K., Tan C.C., Garg S., Zhou X.F. (2017). Lipid-based nanosystem of edaravone: Development, optimization, characterization and in vitro/in vivo evaluation. Drug Deliv..

[30] Thomas J., Carson C.F., Peterson G.M., Walton S.F., Hammer K.A., Naunton M., Davey R.C., Spelman T., Dettwiller P., Kyle G. (2016). Therapeutic potential of tea tree oil for scabies. Am. J. Trop. Med. Hyg..

[31] Rutherford T., Nixon R., Tam M., Tate B. (2007). Allergy to tea tree oil: Retrospective review of 41 cases with positive patch tests over 4.5 years. Australas J. Derm..

[32] Tisserand R. Challenges facing essential oil therapy: Proof of safety. Proceedings of the Alliance of International Aromatherapists (AIA) Conference.

[33] Parikh A., Kathawala K., Song Y., Zhou X.F., Garg S. (2018). Curcumin-loaded self-nanomicellizing solid dispersion system: Part i: Development, optimization, characterization, and oral bioavailability. Drug Deliv. Transl. Res..

[34] Parikh A., Kathawala K., Tan C.C., Garg S., Zhou X.F. (2018). Self-nanomicellizing solid dispersion of edaravone: Part i—Oral bioavailability improvement. Drug Des. Dev. Ther..

[35] Parikh A., Kathawala K., Tan C.C., Garg S., Zhou X.F. (2016). Development of a novel oral delivery system of edaravone for enhancing bioavailability. Int. J. Pharm..

[36] FDA (2019). Inactive Ingredient Search for Approved Drug Products.

[37] Niazi S.K. (2004). Handbook of pharmaceutical manufacturing formulations. Liquid Products.

[38] Masoumi H.R., Basri M., Samiun W.S., Izadiyan Z., Lim C.J. (2015). Enhancement of encapsulation efficiency of nanoemulsion-containing aripiprazole for the treatment of schizophrenia using mixture experimental design. Int. J. Nanomed..

[39] Rao S., Song Y., Peddie F., Evans A.M. (2011). Particle size reduction to the nanometer range: A promising approach to improve buccal absorption of poorly water-soluble drugs. Int. J. Nanomed..

[40] Franz T.J., Lehman P.A., Franz S.F., North-Root H., Demetrulias J.L., Kelling C.K., Moloney S.J., Gettings S.D. (1993). Percutaneous penetration of n-nitrosodiethanolamine through human skin (in vitro): Comparison of finite and infinite dose applications from cosmetic vehicles. Fundam. Appl. Toxicol..

[41] Haidari H., Kopecki Z., Bright R., Cowin A.J., Garg S., Goswami N., Vasilev K. (2020). Ultrasmall agnp-impregnated biocompatible hydrogel with highly effective biofilm elimination properties. ACS Appl. Mater. Interfaces.

[42] Haidari H., Goswami N., Bright R., Kopecki Z., Cowin A.J., Garg S., Vasilev K. (2019). The interplay between size and valence state on the antibacterial activity of sub-10 nm silver nanoparticles. Nanoscale Adv..

[43] Osterberg R.E., See N.A. (2003). Toxicity of excipients--a Food and Drug Administration perspective. Int. J. Toxicol..

[44] National Toxicology Program (1992). Ntp toxicology and carcinogenesis studies of polysorbate 80 (cas no. 9005-65-6) in f344/n rats and b6c3f1 mice (feed studies). Natl. Toxicol. Program Tech. Rep. Ser..

[45] Lee S.G., Kang J.B., Kim S.R., Kim C.J., Yeom D.W., Yoon H.Y., Kwak S.S., Choi Y.W. (2016). Enhanced topical delivery of tacrolimus by a carbomer hydrogel formulation with transcutol p. Drug Dev. Ind. Pharm..

[46] Hebert A., Friedlander S., Allen D.B. (2006). Topical fluticasone propionate lotion does not cause hpa axis suppression. J. Pediatr..

[47] Eichenfield L.F., Miller B.H., Cutivate Lotion Study Group (2006). Two randomized, double-blind, placebo-controlled studies of fluticasone propionate lotion 0.05% for the treatment of atopic dermatitis in subjects from 3 months of age. J. Am. Acad. Derm..

[48] Contri R.V., Frank L.A., Kaiser M., Pohlmann A.R., Guterres S.S. (2014). The use of nanoencapsulation to decrease human skin irritation caused by capsaicinoids. Int. J. Nanomed..

[49] Danaei M., Dehghankhold M., Ataei S., Hasanzadeh Davarani F., Javanmard R., Dokhani A., Khorasani S., Mozafari M.R. (2018). Impact of particle size and polydispersity index on the clinical applications of lipidic nanocarrier systems. Pharmaceutics.

[50] Flores F.C., Ribeiro R., Ourique A., Rolim C., Silva C., Pohlmann A., Beck R., Stanisçuaski Guterres S. (2010). Nanostructured systems containing an essential oil: Protection against volatilization. Química Nova.

[51] Le Ouay B., Stellacci F. (2015). Antibacterial activity of silver nanoparticles: A surface science insight. Nano Today.

[52] Toutain-Kidd C.M., Kadivar S.C., Bramante C.T., Bobin S.A., Zegans M.E. (2009). Polysorbate 80 inhibition of pseudomonas aeruginosa biofilm formation and its cleavage by the secreted lipase lipa. Antimicrob. Agents Chemother..

[53] Nielsen C.K., Kjems J., Mygind T., Snabe T., Meyer R.L. (2016). Effects of tween 80 on growth and biofilm formation in laboratory media. Front. Microbiol..

