# Supplementary material for: Quality-by-Design Approach for the Development of Nano-Sized Tea Tree Oil Formulation-Impregnated Biocompatible Gel with Antimicrobial Properties
Source: Pharmaceutics. 2020 Nov 13;12(11):1091. doi: 10.3390/pharmaceutics12111091 (PMC7698170; doi:10.3390/pharmaceutics12111091)
Supplement: Supplementary file 1 [file pharmaceutics-12-01091-s001.pdf]

pharmaceutics

Supplementary Materials: Quality-by-Design Approach for the Development of Nano-Sized Tea Tree Oil Formulation-Impregnated Biocompatible Gel with Antimicrobial Properties

Thabata Muta, Ankit Parikh, Krishna Kathawala, Hanif Haidari, Yunmei Song, Jackson Thomas and Sanjay Garg

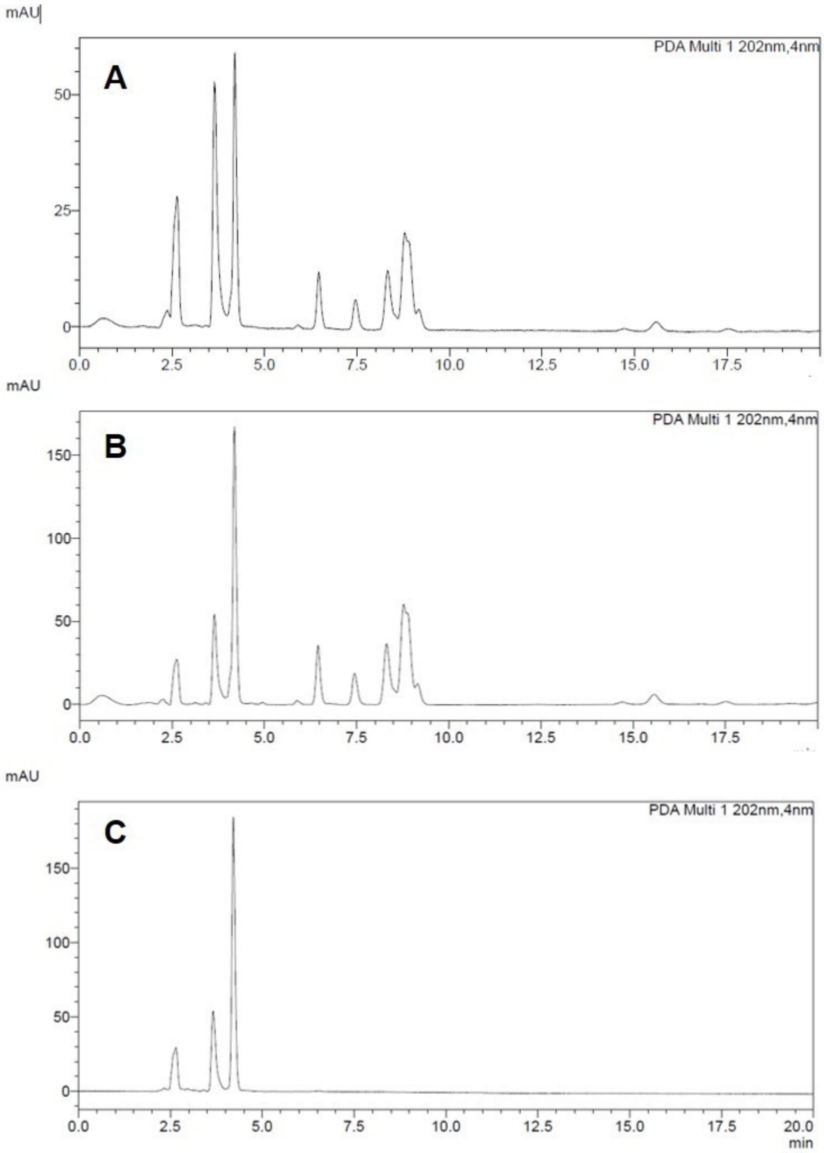

Figure S1. PDA-HPLC chromatograms of TTO and primary reference standard (Terpinen-4-ol). Chromatogram for TTO at concentrations 50 μg/mL (A) and 100 μg/mL (B). Primary reference standard (Terpinen-4-ol) at 50 μg/mL (C), Retention time at 4.2 min.

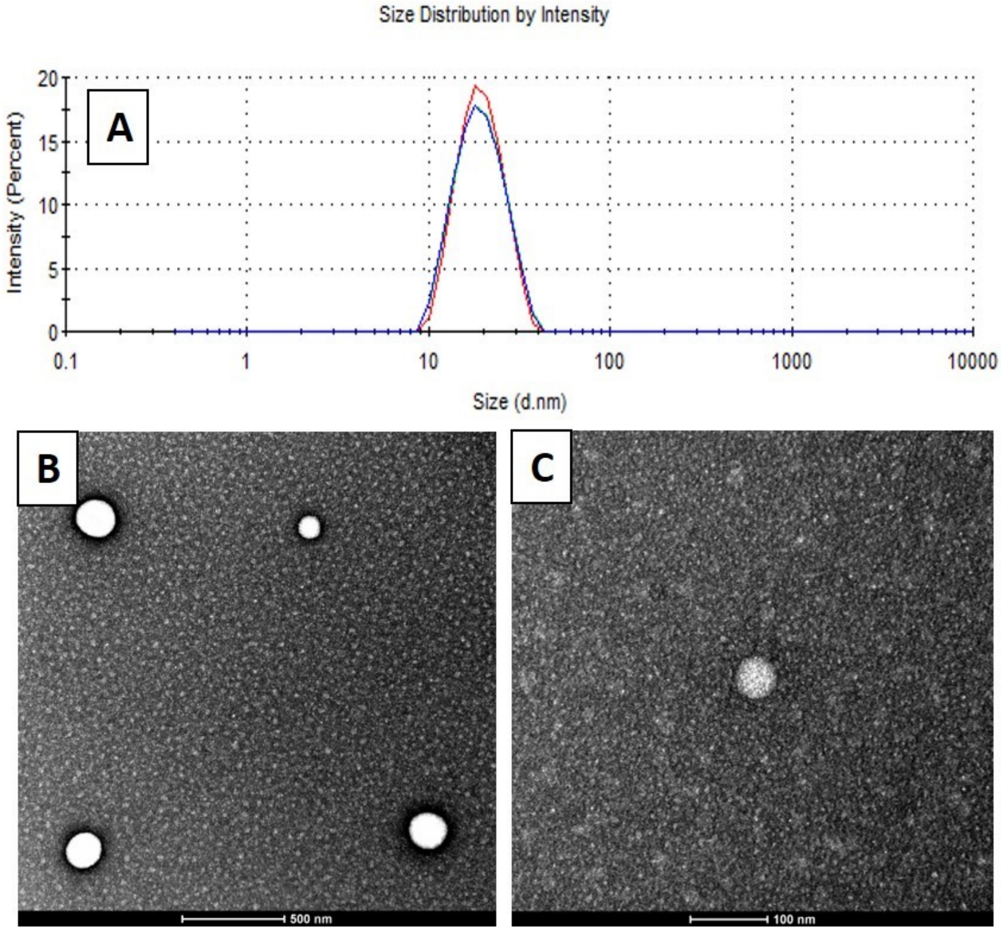

Figure S2. Characterization of optimized TTO-LNF formulation droplet size using zeta sizer (A), transmission electron microscopy with 500 nm scale (B), and 100 nm scale (C).

Table S1. Preliminary screening of components for development of TTO-LNF.

Formulation Code	Propylene Glycol (% w/v)	Tween 80 (% w/v)	Span 80 (% w/v)	Ethanol (% w/v)	Transcutol P (% w/v)	Kolliphor™ RH 40 (% w/v)	TTO (% w/v)	Water (% w/v)	Observation	Droplet size	PDI
TTO001TF001[15]	15	30	-	-	-	-	5	50	Clear	13.57	0.257
TTO001TF002[14]	-	13.68	5.32	19	-	-	5	57	Cloudy	-	-
TTO001TF003[15, 29]	-	-	-	-	15	30	5	50	Clear	17.31	0.063
TTO001TF004	-	30	-	-	15	-	5	50	Clear	16.26	0.306
TTO001TF005	15	-	-	-	-	30	5	50	Clear	27.09	0.198
TTO001TF006	-	30	-	-	30	30	10	-	Clear	-	-
TTO001TF007	-	25	-	-	30	25	20	-	Cloudy	-	-
TTO001TF008	-	20	-	-	30	20	30	-	Cloudy	-	-
TTO001TF009	-	25	-	-	10	25	40	-	Semi-solid	-	-
TTO001TF010	-	25	-	-	20	25	30	-	Semi-solid	-	-
TTO001TF011	-	27.5	-	-	30	27.5	15	-	Cloudy	41.08	0.885
**Formulation Code**	**Propylene Glycol (% w/v)**	**Tween 80 (% w/v)**	**Span 80 (% w/v)**	**Ethanol (% w/v)**	**Transcutol P (% w/v)**	**Kolliphor™ RH 40 (% w/v)**	**TTO (% w/v)**	**Water (% w/v)**	**Observation**	**Droplet size**	**PDI**
TTO001TF012	-	22.5	-	-	30	22.5	25	-	Cloudy	-	-
TTO001TF013	2.385		4.75	2.55		14.3	5	71	Partially Cloudy	-	-
TTO001TF014	-	8.75	-	-	12	8.75	5	65.5	Clear	-	-
TTO001TF015	-	6.25	-	-	12	6.25	5	75	Clear	-	-
TTO001TF016	-	7.5	-	-	5	7.5	5	75	Clear	-	-
TTO001TF017	-	10	-	-	5	10	5	70	Clear	-	-
TTO001TF018	-	7.5	-	-	12	7.5	5	68	Cloudy	-	-
TTO001TF019	-	5	-	-	5	5	5	80	Cloudy	-	-
TTO001TF020	-	10	-	-	10	10	5	65	Clear	-	-
TTO001TF021	-	-	-	-	12	17.5	5	65.5	Cloudy	-	-
TTO001TF022	-	-	-	-	5	15	5	75	Cloudy	-	-
TTO001TF023	-	-	-	-	5	20	5	70	Clear	-	-

pharmaceutics

MDPI

Table S2. ANOVA for droplet size and Cubic Model.

Source	Sum of Squares	df	Mean Square	F-value	P-value
Model	62031.88527	9	6892.432	33.51481	0.002056
Linear Mixture	36873.41019	2	18436.71	89.64943	0.000476
AB	74.8339368	1	74.83394	0.363884	0.578892
AC	58.35335113	1	58.35335	0.283746	0.622473
BC	476.6443327	1	476.6443	2.317708	0.202568
ABC	116.2324584	1	116.2325	0.565186	0.493995
AB(A-B)	177.416901	1	177.4169	0.862699	0.405559
AC(A-C)	41.81703235	1	41.81703	0.203337	0.675404
BC(B-C)	519.561834	1	519.5618	2.526396	0.187158
Residual	822.6133334	4	205.6533		
Cor Total	62854,49861	13			

A, Surfactant (Tween 80 and Kolliphor™ RH40, w/w, 50/50); B, Co-surfactant (Transcutol P); C, water.

Table S3. ANOVA for PDI and Cubic Model.

Source	Sum of Squares	df	Mean Square	F-value	P-value
Model	0.210834064	9	0.023426	23.04619	0.004238
Linear Mixture	0.172370242	2	0.086185	84.78774	0.000531
AB	0.000561668	1	0.000562	0.552561	0.498562
AC	0.00072127	1	0.000721	0.709575	0.447004
BC	0.009885085	1	0.009885	9.724811	0.035579
ABC	0.000674689	1	0.000675	0.66375	0.460964
AB(A-B)	0.000595032	1	0.000595	0.585385	0.486857
AC(A-C)	0.00122033	1	0.00122	1.200544	0.334756
BC(B-C)	4.00529E-05	1	4.01E-05	0.039404	0.852332
Residual	0.004065924	4	0.001016		
Cor Total	0.214899988	13			

A, Surfactant (Tween 80 and Kolliphor™ RH40, w/w, 50/50); B, Co-surfactant (Transcutol P); C, water.
